# Honey exposure stimulates wound repair of human dermal fibroblasts

**DOI:** 10.4103/2321-3868.113333

**Published:** 2013-06-18

**Authors:** Elia Ranzato, Simona Martinotti, Bruno Burlando

**Affiliations:** Dipartimento di Scienze e Innovazione Tecnologica, DiSIT, University of Piemonte Orientale “Amedeo Avogadro”, viale T. Michel 11, 15121 Alessandria, Italy

**Keywords:** Honey, wound healing, interleukins, dermal fibroblast

## Abstract

Honey is widely used for treating burns, ulcers and wounds, but the mechanisms of action are poorly known and the product is mainly used as an antimicrobial. We have examined here the wound healing properties of honey on human fibroblasts, using an *in vitro* scratch wound healing model. Three kinds of widely used monofloral honeys were used, viz. acacia (*Robinia pseudacacia*), buckwheat (*Fagopyrum sp.*), and manuka (*Leptospermum scoparium*). Data displayed an increased wound healing activity in fibroblasts, but with different efficiency and mechanisms of action among honeys. The effects of acacia and buckwheat emerged in both scratch wound and chemotaxis assays, while the effect of manuka was significant but lower. The use of inhibitors indicated on the whole an essential role of cytosolic calcium, an important role of ERK and p38, and a secondary role of PI3K. Acacia and buckwheat, but not manuka, induced significant increases in the release of interleukin-4 (IL-4), IL-6, and IL-8, indicating a correlation between interleukin upregulation and wound closure efficiency. This is consistent with our previous findings suggesting a higher ability of acacia and buckwheat to activate keratinocyte reepithelialization, with respect to manuka honey. In conclusion, our data indicate that acacia and buckwheat honeys are particularly efficient in facilitating fibroblast wound closure activities, suggesting new therapeutic possibilities for this natural product.

## Introduction

Honey has a long and interesting history. Since Biblical times and before, it has been known to have beneficial health effects, and is still widely used in ‘folk medicine’. Recently, the therapeutic virtues of honey have been rediscovered by the medical profession and are gaining acceptance for treating ulcers, wounds and other surface infections. Literature reports show that honey has been successfully used on infections not responding to standard antiseptic and antibiotic therapy.[1,2]

**Table:** 

Access this article online	
**Quick Response Code**:	**Website**: www.burnstrauma.com
	**DOI**: 10.4103/2321-3868.113333

In spite of an increasing diffusion of honey for clinical uses, the subjacent mechanisms of action are still largely obscure. However, it has been already shown that the usefulness of honey in treating wounds is not limited to its antibacterial action. For instance, honey promotes rapid growth of healthy granulation tissue and acts as an anti-inflammatory agent.[1] Zumla *et al.*,[3] briefly reviewed the use of honey for therapeutic purposes, and from the widespread reports of clinical uses, they concluded that it was time for honey to receive due recognition by the medical world.

Wound healing is a complex process entailing several interactions among cells, the extracellular matrix, and factors involved in inflammatory responses and vascular repair.[4] The process is generally divided into three parts, known as inflammatory, proliferation and maturation phases.[5] The inflammatory phase, usually lasting up to 4 days, is dominated by platelet accumulation at the wounded sites, followed by immune cell migration into the wound, especially neutrophils and macrophages. The proliferative phase is characterized by the arrival and proliferation of fibroblasts that synthesize a provisional collagen type III matrix (granulation tissue) and differentiate into myofibroblasts allowing wound contraction. During this phase, keratinocytes also migrate from wound edges and reform the upper epidermis. The maturation phase involves the remodeling of collagen from type III to type I, operated by fibroblasts, in association with the apoptosis of myofibroblasts, endothelial cells and macrophages. This phase can last in some cases for one year or longer.

Honey is increasingly used as a wound dressing in clinical settings,[6] but the relationships between its healing properties and the wound repair mechanism are still largely unexplored.[7] Therefore, this study was designed to investigate the potential wound healing effects of honey on fibroblasts. We used a well-assessed *in vitro* scratch wound healing model consisting of human fibroblasts.[8,9] *In vitro* tests are now widely employed in pharmacological research because of ethical reasons and due to their usefulness in bioactive-guided fractionation and determination of active compounds.[10]

We used three kinds of widely used honeys, viz. acacia, buckwheat, and manuka. Acacia honey (black locust, *Robinia pseudacacia*) has been experimentally documented for its wound-healing activity on rat models.[11] Buckwheat honey (genus *Fagopyrum*) is a polyphenol-rich dark honey with well-known antioxidant and anti-inflammatory properties.[12] Manuka honey (New Zealand tea tree, *Leptospermum scoparium*) has antibacterial and healing activities, useful for treating problematic wounds.[13] Our data showed an increased wound healing activity in fibroblasts exposed to honey, but with differential efficiency and mechanism of action among different honey types.

## Materials and methods

### Honeys

Samples of acacia, buckwheat, and manuka honeys were obtained from Yamada Apiculture Center, Inc. (Tomata-Gun, Okayama, Japan).

### Cell culture and reagents

All reagents were from Sigma-Aldrich, unless otherwise indicated. Human fibroblast cell line (46 BR.1N) was obtained from European Collection of Cell Cultures (ECACC). The cell line was originally derived from the skin of an individual with hypo-gammaglobulinemia, and immortalized by transfection with the plasmid pSV3neo, expressing SV40 T-antigen.*[31]* Cells were maintained at 37°C, 5% CO2, in DMEM supplemented with 10% fetal bovine serum (FBS) and 1% antibiotic mixture.

### Calcein-acetoxymethylester (Calcein-AM) assay

The lipophilic, nonfluorescent Calcein-AM penetrates cell membranes and is then cleaved by intracellular esterases, yielding the hydrophilic fluorescent dye. Cells were settled overnight in 96-well plates (8,000 cells/well), incubated with honey for 24 h, washed with PBS, and then incubated for 30 min at 37°C with a solution of 2.5 μM calcein-AM in PBS. Plates were read in a fluorescence plate reader (Infinite 200 Pro, Tecan, Wien, Austria), by using 485-nm exc and 535-nm em filters.

### Scratch wound assay

Fibroblasts were settled in 12-well plates and grown to confluence. Thereafter, scratch wounds were created in cell monolayers by using a sterile 0.1-10 μL pipette tip. After washing away suspended cells, cultures were refed with medium in the presence of different concentrations of honey for 24 h. Cells were then fixed in 3.7% formaldehyde in PBS for 30 min, and then stained with 0.1% toluidine blue at room temperature for 30 min. The width of the wound space was measured at wounding and at the end of treatments, using an inverted microscope equipped with a digital camera (Leica Microsystems). Digitized pictures of wounds were analyzed using the NIH Image J software. In a typical experiment, each group consisted of three different plates, i.e. a total of six wounds. Four measurements of wound width were made for each wound at randomly chosen points. Measurements were made by a single observer unaware of the treatments. Wound closure rates were determined as the difference between wound width at 0 and 24 h.

### Cell migration assay

A cell migration assay was performed in transwell plates (8 μm pore size, Costar, Cambridge, MA). A total of 1×10^5^ cells per well were seeded in the upper compartment of filters, while medium containing 0.1% honey was put in the lower compartment. After 24 h filters were removed and stained for 10 min with 0.5% crystal violet (145 mM NaCl, 0.5% formal saline, 50% ethanol), and then washed thrice with water. The upper side of filters was scraped using a cotton swab to remove cells that had attached but not migrated. Following PBS washing of filters, the dye was eluted from cells with 33% acetic acid, and measured at 540 nm in a plate reader (Infinite 200 Pro, Tecan, Wien, Austria). Chemotaxis was assessed by analyzing five independent filters.

### Matrix metalloproteinase antibody array

Matrix metalloproteinases and their tissue inhibitors (MMP−1, −2, −3, −8, −9, −10, −13, and TIMP−1, −2, −3, −4) were determined in cell culture supernatants by an antibody array kit (RayBio MMP antibody array 1, RayBiotech, Norcross, GA) following the manufacturer’s protocol. The array consists of highly specific and well-characterized antibodies spotted on a nitrocellulose membrane. Cells were grown for 24 h in the presence of honey and conditioned media were then collected. Detection membranes were blocked with blocking buffer for 1 h at room temperature (RT) and then incubated with conditioned media. Membranes were washed, incubated with 1 ml of primary biotin-conjugated antibody at RT for 2 h, washed, incubated with 2 ml of horseradish peroxidase-conjugated streptavidin at RT, and then developed using enhanced-chemiluminescence solution (ECL), provided in the kit. Spots were observed, digitized and quantified with the ChemiDoc XRS system, using Quantity One Imaging software (Bio-Rad Laboratories, Hercules, CA).

### Cytokine antibody array

Cytokines were quantified using the Human Cytokine Antibody Array kit 1.0 (Panomics, Inc., Redwood City, CA). The array (see above) allows for simultaneous detection of 18 cytokines and provides positive and negative controls. Cells were seeded in 12-well plates for 24 h, and then exposed to honey for 24 h. Collected conditioned media were then incubated for 1.5 h with membrane-immobilized capture antibodies specific to a particular cytokine protein. Unbound proteins were washed away. A second, biotin-conjugated antibody was allowed to bind for 1.5 h to a second epitope on the protein. Thereafter, 1 h incubation with streptavidin-horseradish peroxidase allowed visualization of proteins through detection of chemiluminescent signal. Spots were quantified as above.

### Statistical analysis

Data were analyzed by ANOVA and the post hoc Tukey’s test, using the Instat software package (GraphPad Software, Inc, San Diego, CA). Median (EC_50_) and minimum (EC_05_) effective concentrations and their 95% confidence intervals were determined by using a downhill logistic dose-response curve developed by CSIRO, Australia:[32]where T=top, S=Hill’s slope (negative for a downhill curve), D=honey concentration (% v/v). Statistical comparisons between EC50 values were based on overlapping or non-overlapping 95% CI.

## Results

### Honey induces fibroblast wound healing

Honey cytotoxicity tests were carried out on fibroblasts in order to optimize the dosages to be used in scratch wound analyses. Calcein-AM assay data showed similar, low cytoxicity levels for all honeys [Table 1]. Based on these data, in subsequent experiments we used honey doses of 0.1% (v/v), a value that is below the EC_05_ of each honey, and has been already used in an analogous study on keratinocytes.[7]

**Table 1:** Values of EC05 and EC50 (% v/v) determined for different honeys by the calcein-AM endpoint at 24 h

	EC_05_	EC_50_
buckwheat	0.47	1.22
	(0.37−0.60)	(1.11−1.35)
manuka	0.35	2.18
	(0.17−0.68)	(1.76−2.70)
acacia	2.28	4.00
	(1.69−3.08)	(3.59−4.46)

Experiments were carried out in triplicate with a minimum of 8 replicates each; 95% CI are given in parentheses

**Figure 1:**
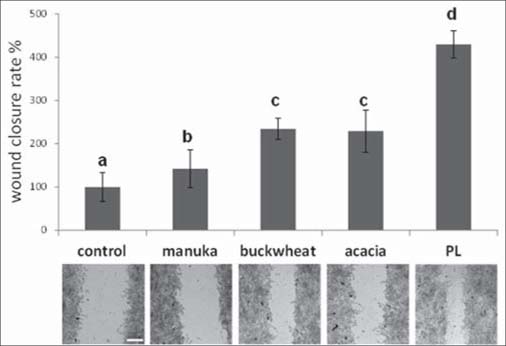
Scratch wound healing of fibroblast confluent monolayers. Cells cultured in 12-well plates were mechanically scratched with a sterile 0.1–10 μL pipette tip, and then allowed to reepithelialize for 24 h at 37°C in the presence of different honeys at 0.1% v/v. One sample was exposed to 20% platelet lysate (PL) as positive control (see text). Upper panel: measurements of wound closure rates expressed as the difference between wound width at 0 and 24 h. Bars represent mean±SD of two independent experiments, each with n = 20. The mean of control has been set to 100. Letters on bars indicate clustering on the base of statistical differences determined by pairwise comparisons with the Tukey’s test. Values labeled with same letters are not statistically different from each other, whereas different letters indicate statistical differences (*P* < 0.01). Lower panel: micrographs of scratch wounded fibroblasts incubated under control conditions, or in the presence of various honeys at 0.1% (v/v), or of 20% PL, and then stained with blue toluidine and observed at 24 h. Scale bar, 200 μm.

Confluent monolayers of fibroblasts were scratch wounded and then were incubated with or without honey. One series of positive controls were exposed to 20% (v/v) of a platelet lysate (PL), which had been previously shown to promote scratch wound healing in these cells.[8] The PL was obtained from blood samples as described in Ranzato *et al.*[8] Cells exposed to buckwheat and acacia honeys showed significantly higher wound closure rates at 24 h with respect to controls, while manuka honey induced a significant but lower effect [Figure 1].

### Honey chemoattractant effect

**Figure 2:**
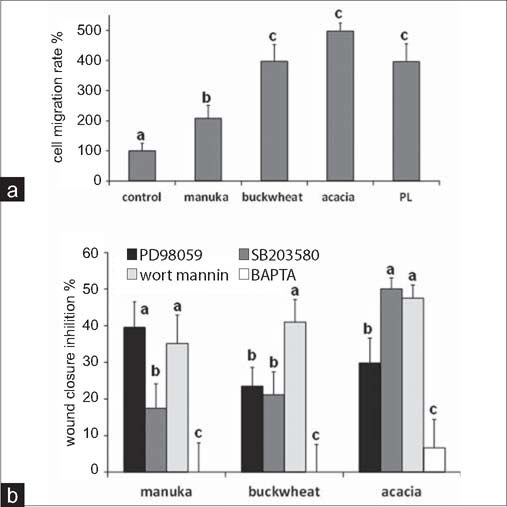
(a) Effect of 0.1% honey, and of 20% PL as positive control, on fibroblast cell migration evaluated by a transwell assay (see Methods). Data are mean±SD (*n*=5) of cell migration rate (see Methods), expressed as percent variation with respect to control. Statistics as in Figure 1. (b) Effect of different inhibitors on honey-induced scratch wound repair of fibroblast monolayers. Data were recorded 24 h after scratch wound healing of cells exposed to 0.1% honey, in the presence or absence of various inhibitors. Bars represent mean±SD of percent wound closure inhibitions recorded in two independent experiments, each with n=20. For each honey type, all groups are significantly different from controls “(100%). Different letters on bars indicate significant differences among different inhibitor groups according to the Tukey’s test (*P* < 0.01). Values labeled with same letters are not statistically different from each other.

To obtain a direct evidence of whether honeys influence cell migration rates, we used a chemotaxis assay. In the presence of 0.1% honey, the number of migrating cells was significantly increased for all honeys with respect to control (*P*<0.01). Acacia honey induced an effect stronger than that of PL, while buckwheat’s effect was similar to PL. The effect of manuka honey was lower but significant [Figure 2a]. These data revealed chemoattractant effects for all honeys, but confirmed the lower potential of manuka with respect to the other two honey types, thus suggesting differences among the mechanisms of action.

### Effects of inhibitors

To investigate the mechanism of action of honey on wound closure, we performed scratch wound experiments at 24 h in the presence of signal transduction pathway inhibitors, such as PD98059 (ERK kinase, 10 μM), SB203580 (p38 kinase, 20 μM), wort mannin (PI3K kinase, 500 nM) and BAPTA-AM (cell-permeant calcium chelator, 30 μM). These inhibitors did not alter the basal wound closure rate in the absence of honey, with the exception of BAPTA (*P* < 0.01). In addition, BAPTA almost completely abolished the healing effect of honeys. The other drugs induced different inhibitory effects on different honey types [Figure 2b]. The wound healing activity of acacia honey showed the lowest reduction, and was maximally sensitive to PD98059. Manuka honey was partially inhibited by wort mannin and PD98059, and strongly inhibited by SB203580, while buckwheat honey was partially inhibited by wort mannin and strongly inhibited by PD98059 and SB203580 [Figure 2b]. The vehicle alone (0.1% DMSO) produced no influence on wound closure, either in the presence or absence of honeys (*P* > 0.05).

### Variations in MMP and cytokine expression

**Figure 3:**
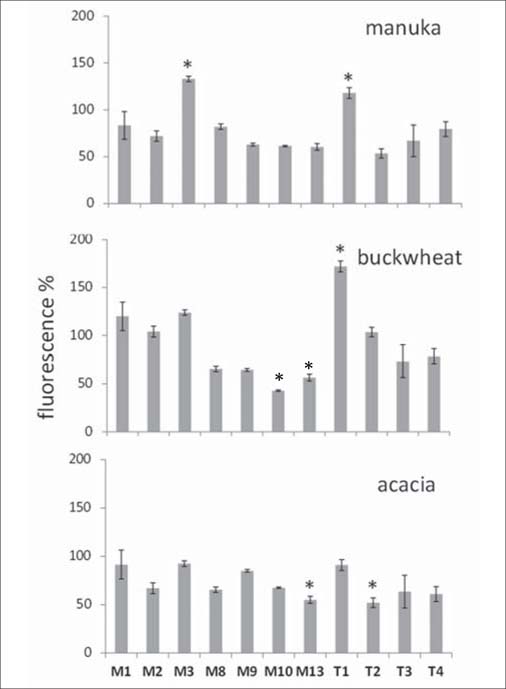
Induction of matrix metalloproteinases (MMPs) after honey exposure. Confluent monolayers of cells were exposed to 0.1% honeys for 24 h. Culture supernatants were harvested and MMPs and their tissue inhibitors (TIMPs) were detected using the MMP-Array kit from RayBiotech, Inc. (see Methods). Bars are mean±SD of fluorescence arbitrary units recorded in two independent experiments, expressed as percent variation respect to control. *P < 0.05, according to Tukey’s test.

Conditioned media collected from fibroblasts exposed to honeys were used to probe for the presence of various MMPs and TIMPs by using an antibody array. By considering significant variations only (*P*<0.05), increases of MMP-3 and TIMP-1 expression were observed with manuka, an increase of TIMP-1 and a decrease of MMP-10 and MMP-13 with buckwheat, and a decrease of MMP-13 and TIMP-2 with acacia. The expression of other MMPs and TIMPs remained substantially unchanged [Figure 3].

**Figure 4:**
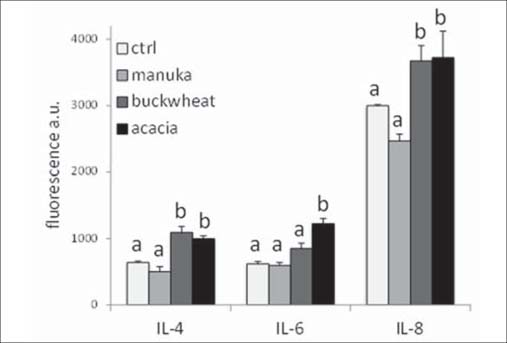
Induction of interleukins after honey exposure. Confluent monolayers of cells were treated as in Figure 3, and thereafter different interleukins were detected using the Cytokine antibody array kit from Panomics Inc. (see Methods). Bars are mean±SD of fluorescence arbitrary units from two independent experiments. For each interleukin type, values labeled with same letters are not statistically different, whereas different letters on bars indicate significant differences among groups according to Tukey’s test (*P* < 0.01).

The same samples were also probed with an antibody array for a series of cytokines. This analysis revealed an abundant presence of interleukin-8 (IL-8), and fairly detectable amounts of IL-4 and IL-6. The expressions of all these interleukins were increased by acacia honey, while those of IL-4 and IL-8 by buckwheat. Conversely, no significant change was detected with manuka [Figure 4].

## Discussion

Our data demonstrated that honey exposure induces low cytotoxicity on fibroblasts, in accordance with previous results on keratinocytes,[7] suggesting that honey can be considered as a safe compound for external applications.

Scratch wound data and cell migration assay showed that honey improves fibroblast wound repair capabilities. Cell motility is a key element of tissue repair processes, and therefore, its induction could explain the ability of honey to promote the scratch wound healing of fibroblasts, similar to what was reported for keratinocytes.[7] Also, the comparison with the platelet lysate shows a stronger effect of honey as a chemoattractant than on wound closure. This could be a therapeutically important feature, because it could favor fibroblast invasion of the wounded site during the proliferative phase.

In order to understand the mechanisms underlying this effect, we used a battery of inhibitors of cell signaling pathways that are known to be directly involved in the wound process.[14] BAPTA-AM was found to be as the most effective inhibitor of scratch wound closure, confirming an essential role of intracellular calcium, as previously shown on these cells[8] and on other cell types.[4,9,15–21] The other inhibitors showed variable effects on different honeys, suggesting the existence of different mechanisms of action. Acacia activated mostly the ERK pathway, buckwheat mostly ERK and p38, and manuka mostly p38, while the least activated pathway was PI3K. A similar pattern of activation of these pathways was found in a previous study concerning the effect of a platelet lysate, and carried out on the same scratch wound model used here.[8] In addition, ERK1/2, p38 and PI3K/Akt were also shown to mediate the stimulation of locomotion and proliferation induced in murine fibroblasts by plate derivative growth factor (PDGF), a main growth factor acting in the early phases of wound healing.[22–24]

Current literature provides evidence that MMPs and their inhibitors are essential in tissue repair. MMPs and TIMPs are known to control the inflammatory phase of wound healing by modulating chemokine and cytokine cleavage, while they also regulate matrix degradation and remodeling.[25] Majtan *et al.*,[26] and Ranzato *et al.*,[7] have consistently demonstrated that honey exposure induces gelatinase MMP-9 expression in human keratinocytes, thus confirming a similar effect obtained on the same cells with the platelet lysate.[4] In contrast, the platelet lysate does not seem to affect gelatinase expression in fibroblasts.[4] This is another point of convergence with the present investigation, where MMP or TIMP upregulation by honey was limited to MMP-3 induction with manuka, and TIMP-1 with manuka and buckwheat. The increase of TIMP-1 upon buckwheat exposure could be linked to the anti-inflammatory effects of this protein, considering that it has been induced by cytokine exposure in dermal fibroblasts.[27]

Cytokines are known to play a role in wound healing, and accordingly, our data provide a close correlation between interleukin modulation and wound healing activity. Acacia and buckwheat honeys, which were most active in promoting scratch wound closure, also induced significant increases of primary interleukins present in our fibroblast culture, viz. IL-4, IL-6, and IL-8. The involvement of all these factors in wound healing has been previously documented.[28,30]

Hence, different kinds of evidence indicate that acacia and buckwheat honeys are more efficient than manuka honey in promoting the tissue repair activity of fibroblasts. Most notably, these data are consistent with our previous findings, suggesting a higher ability of acacia and buckwheat to activate keratinocyte re-epithelialization.[7]

In summary, our present and previous findings consistently indicate that honey is generally active in facilitating wound closure, thus confirming a bulk of anecdotal and scientific evidence. However, the novelty of our data consists in showing that different types of honey act with different mechanisms, and moreover that some of these mechanisms are more efficient than others. In particular, acacia and buckwheat honeys have shown more powerful wound-healing promoting activities with respect to manuka honey on both dermal and epidermal cells. Hence, our data indicate that the complex of different honey types offers a wider set of therapeutic possibilities, raising new medical interest in this valuable natural product.
